# Performance Study of Asphalt Mixtures Reinforced with Gradated Basalt Fibers of Mixed Lengths

**DOI:** 10.3390/ma17194706

**Published:** 2024-09-25

**Authors:** Xiaoxiang Ji, Yuqing Yuan, Yunjun Huang, Jinggan Shao, Sihao Li

**Affiliations:** 1 School of Highway, Henan College of Transportation, Zhengzhou 451450, China; docling@126.com (X.J.); sjgjygs@163.com (J.S.); 2 School of Civil Engineering and Architecture, Henan University, Kaifeng 475004, China; lisihao2023@126.com; 3 Henan Jiaoyuan Engineering Group Technology Co., Ltd., Zhengzhou 451450, China; hyj8293@163.com

**Keywords:** road engineering, basalt fibers, asphalt mixtures, gradation, grey target decision method

## Abstract

The length of basalt fibers affects the performance of asphalt mixtures. To explore the influence of different lengths of basalt fibers on the performance of asphalt mixtures, this study selected basalt fibers with lengths of 6 mm, 9 mm, and 12 mm to design gradations that were incorporated into asphalt mixtures to prepare specimens. High-temperature rutting tests, immersion Marshall tests, freeze-thaw splitting tests, and low-temperature splitting tests were conducted, resulting in 11 test mix designs and 12 test indicators. Then, a multi-objective grey target decision method was used to optimize the optimal combination ratio of basalt fiber lengths. The results indicate that compared to asphalt mixtures with single-length basalt fibers, incorporating well-combined basalt fibers significantly enhances the high-temperature, low-temperature, and water stability performance of asphalt mixtures. According to the grey target decision method, this study determined that a basalt fiber combination ratio of 3:4:3 for lengths of 6 mm, 9 mm, and 12 mm provides the best overall performance of asphalt mixtures. Additionally, when designing asphalt mixtures with graded basalt fibers, the inclusion of 9 mm fibers should be the primary control point. These research findings provide important guidance for the enhanced application of basalt fibers in road engineering.

## 1. Introduction

Asphalt concrete pavement is one of the most commonly used road surfaces today, known for its high degree of mechanization in construction and maintenance, quick traffic accessibility, comfortable driving experience, and low noise levels. However, due to various complex factors, the pavement is prone to distress such as cracks and rutting [1,2], making its performance under harsh conditions like high and low temperatures a key focus in the engineering community [3,4,5].

To enhance the performance of asphalt concrete, researchers have explored various fiber-reinforced materials, including lignin fiber, synthetic polyester fiber, glass fiber, and basalt fiber. Basalt fiber, in particular, has garnered attention due to its excellent mechanical properties and durability [6,7,8,9]. Basalt fiber is a new type of environmentally friendly mineral fiber produced from basalt rock, known for its high strength, high-temperature resistance, and corrosion resistance, making it one of the natural materials for enhancing asphalt concrete performance [10,11,12]. Basalt fiber can envelop asphalt binder together with coarse aggregates, forming a good interlocking structure that effectively improves asphalt pavement’s resistance to high-temperature rutting [13,14,15], low-temperature cracking [16,17,18], fatigue resistance [19,20], freeze-thaw durability, and water damage resistance [21,22].

Relative to lignin and polyester fibers, the primary purpose of incorporating basalt fibers is to control deformation and enhance the matrix toughness through fiber bonding [17]. Basalt fibers form a stable three-dimensional network in asphalt mortar, exhibiting excellent overall properties, with their interlocking network being a major contributor to improved asphalt mixture performance [13]. The length and diameter of basalt fibers are crucial parameters [23,24], with fiber length being a focal point for researchers. Zhang utilized digital image processing to determine the impact of fiber length distribution on the cracking resistance of basalt fiber asphalt mixtures [25]. Typically, fibers ranging from 3 mm to 15 mm are used, with certain lengths showing relatively satisfactory enhancement capabilities as validated by Zhang’s 3D numerical simulation study [26]. Regarding the influence of basalt fibers on asphalt mixture fracture toughness, Pirmohammad, S. suggested that a fiber length of 4 mm is most suitable [27]. Xie, T. et al., focusing on the rheological properties of basalt fiber-modified asphalt mortar, proposed that a fiber length of 6–9 mm offers optimal overall performance [28]. Wang, W. et al., utilizing response surface methodology (RSM), mesh basket drainage tests, and static probe tests, identified 6 mm as the optimal fiber length for basalt fibers [29], a conclusion also reached by Xu, J. and Guo, F. [30,31]. Lou, K. noted that the optimal fiber length is significantly influenced by the Nominal Maximum Aggregate Size (NMAS) of hot mix asphalt, recommending longer fiber lengths (6 mm to 12 mm) for mixtures with larger NMAS values [23]. It is evident that researchers have differing conclusions regarding the optimal length of basalt fibers. However, it is clear that basalt fiber length has a significant impact on mixture performance, with experimental studies typically employing fiber lengths ranging from 3 mm to 15 mm [23,25,27,29].

Due to the gaps between coarse and fine aggregates in basalt fiber mixtures, which vary in size from millimeters to centimeters and are uneven, these are typically filled with a binder composed of basalt fibers, asphalt, fine aggregates, and mineral powder. Different gradations directly influence the properties of the mixture. Regarding the voids within the mixture, different lengths of basalt fibers working together may better fill these voids compared to single-length fibers [23,25]. Currently, most studies focus on the effects of fiber content, fiber type, and single fiber length on asphalt mixture performance, with fewer studies investigating the optimal grading of basalt fibers of different lengths.

Therefore, addressing the challenge of selecting the optimal mix of basalt fibers due to multiple factors, this study utilizes three lengths (6 mm, 9 mm, and 12 mm) of basalt fibers blended together in proportions to form a fiber gradation. These fibers are incorporated into asphalt mixtures to prepare specimens for high-temperature rutting tests, immersion Marshall tests, freeze-thaw splitting tests, and low-temperature splitting tests. These tests investigate the high-temperature rut resistance and dynamic stability, immersion residual stability, freeze-thaw splitting strength, low-temperature tensile strength, and modulus of rupture changes of the asphalt mixtures. Based on 11 formulated experimental mix designs and 12 performance indicators of the mixtures obtained from experiments, a matrix was constructed and analyzed using the grey target decision-making method [32,33,34,35,36]. The optimal basalt fiber gradation scheme was determined, providing a multi-objective and multi-influence factor decision-making method for the design of fiber-modified asphalt mixtures in engineering practice.

## 2. Materials and Methods

### 2.1. Raw Materials

#### 2.1.1. Asphalt

No. 70 asphalt was chosen as the base asphalt, with technical specifications listed in Table 1.

#### 2.1.2. Aggregates

(1) Coarse and fine aggregates

Both coarse and fine aggregates used in the tests are limestone. Technical specifications are provided in Table 2.

(2) Mineral Powder

The mineral powder is produced by grinding limestone, with technical specifications provided in Table 3.

#### 2.1.3. Fibers

**Figure 1:**
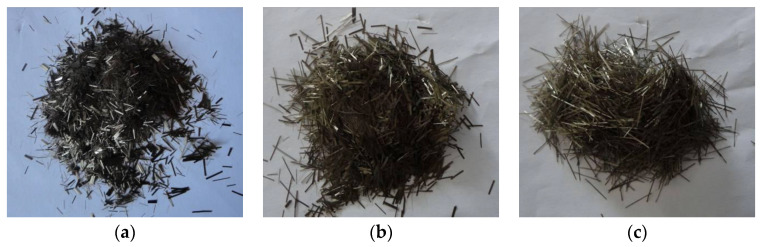
Short-cut basalt fiber: (**a**) 6 mm; (**b**) 9 mm; (**c**) 12 mm.

Basalt fibers used are short-cut basalt fibers with lengths of 6 mm, 9 mm, and 12 mm (Figure 1). Technical indicators are provided in Table 4.

#### 2.1.4. Asphalt Mixture Design

**Figure 2:**
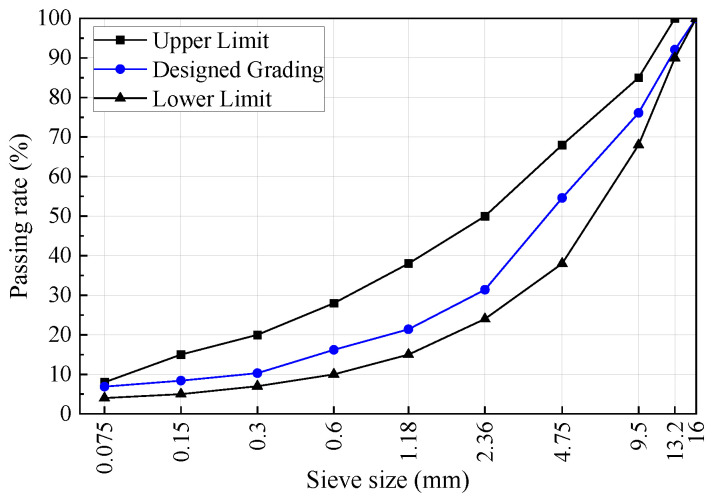
Design gradation of asphalt mixture ratio.

Following the testing methods outlined in JTG E20-2011 [37], and considering the technical requirements specified in JTG F40-2004 [38], Marshall specimens were prepared. Based on criteria such as voids in the mineral aggregate (VMA) and voids filled with asphalt (VFA), the asphalt mixture gradation design is presented in Figure 2. The optimal asphalt-to-aggregate ratios for AC-13 asphalt mixtures with and without basalt fiber additions are determined to be 4.4% and 4.2%, respectively.

#### 2.1.5. Fiber Gradation Scheme Design

Based on the asphalt mixture design gradation specified in Table 5, 0.4% basalt fiber is added with lengths of 6 mm, 9 mm, and 12 mm. The blending ratios for different fiber lengths are determined according to Table 5. S0 in Table 5 represents the blank control scheme with no added fibers.

### 2.2. Experimental Methods

#### 2.2.1. Rutting Test

The rutting test is a method used to evaluate pavement performance by simulating the pressure and strain generated by vehicles traveling over the pavement, thus assessing the pavement’s load-bearing capacity and durability. First, rutting test specimens with dimensions of 300 mm × 300 mm × 50 mm are prepared using a HYCX-1 rutting specimen molding machine (roller compaction line load of 300 N/cm), made by Hangton Test Instruments, Beijing, China. The specimens are cured at room temperature for at least 12 h. Next, the specimens, along with the molds, are placed in a constant temperature chamber set to 60 °C ± 1 °C, where they are maintained for a period ranging from 5 to 24 h. Finally, the specimens are demolded and placed in an HYCZ-5C automatic rutting tester (with a rolling speed of 42 cycles/min ± 1 cycle/min), made by Hangton Test Instruments, Beijing, China. During the rutting test, a solid rubber tire with a wheel pressure of 0.7 MPa rolls over the rutting plate at a speed of 42 cycles/min. After the test, the dynamic stability is calculated using Formula (1) [37].

(1) $$DS=\frac{\left(t_{2}-t_{1}\right)\times N}{d_{2}-d_{1}}\times C_{1}\times C_{2}$$

where *DS* represents the dynamic stability of the asphalt mixture, cycles/mm; *d*_1_ is deformation at time *t*_1_ (typically 45 min), in mm; d_2_ is deformation at time *t*_2_ (typically 60 min), mm; *C*_1_ is the correction factor for the testing instrument; *C*_2_ is the correction factor for the specimen; *N* is the number of wheel passes per minute, cycles/min.

#### 2.2.2. Immersion Marshall Test

The immersion Marshall stability test is used to evaluate the resistance of asphalt mixtures to stripping when subjected to water damage. First, Marshall specimens are molded using the standard compaction method with dimensions of 101.6 mm ± 0.2 mm in diameter and 63.5 mm ± 1.3 mm in height. Then, one set of specimens is placed in a constant temperature water bath at 60 °C for 30 min, followed by testing for stability (*MS*) using a SYD-0709A automatic Marshall testing machine (maximum load of 50 kN), manufactured by Changji Geological Instrument, Shanghai, China. Another set of specimens is kept in the 60 °C water bath for 48 h before testing for stability (*MS*_1_). Finally, the water immersion residual stability of the specimens is calculated using Formula (2) [37].

(2) $$MS_{0}=\frac{MS_{1}}{MS}\times 100$$

where *MS*_0_ represents the residual stability of the specimen after immersion, %; *MS* is the stability of the Marshall specimen; and *MS*_1_ is the stability of the specimen after 48 h of immersion, kN.

#### 2.2.3. Freeze-Thaw Splitting Test

The freeze-thaw splitting test involves subjecting asphalt mixtures to freeze-thaw cycles under specified conditions and measuring the strength ratio of the specimens before and after water damage to analyze the water stability of the asphalt mixture. First, prepare Marshall specimens according to the described testing methods. Next, store one set of specimens at room temperature for future use, and freeze the other set at −18 °C for 16 h, then immerse them in hot water at 60 °C for 24 h. Afterward, condition both sets of specimens in a constant-temperature water bath at 25 °C for 2 h. Finally, perform the splitting test using a UTM-30 material testing machine (static loading ±30 kN) with a loading rate of 50 mm/min, made by IPC Global, GA, USA. The freeze-thaw splitting strength ratio is calculated using Formulas (3)–(5) [37].

(3) $$R_{T1}=0.00628p_{T1}/h_{1}$$

(4) $$R_{T2}=0.00628p_{T2}/h_{2}$$

(5) $$TSR=\frac{{\bar{R}}_{T2}}{{\bar{R}}_{T1}}\;\times \;100$$

where *R_T_*_1_ is the splitting tensile strength of a single specimen from the first group that did not undergo freeze-thaw cycling, MPa. *R_T_*_2_ is the splitting tensile strength of a single specimen from the second group that underwent freeze-thaw cycling, measured in megapascals MPa. *P_T_*_1_ and *P_T_*_2_ are the test load values for single specimens from the first and second groups, respectively, N. *h*_1_ and *h*_2_ are the heights of single specimens from the first and second groups, respectively, mm. *TSR* is the freeze-thaw splitting strength ratio, %. $\overset{-}{R}$*_T_*_2_ is the average splitting tensile strength of the effective specimens from the second group after freeze-thaw cycling, MPa. $\overset{-}{R}$*_T_*_1_ is the average splitting tensile strength of the effective specimens from the first group that did not undergo freeze-thaw cycling, MPa.

#### 2.2.4. Low-Temperature Splitting Test

The low-temperature splitting test is used to determine the mechanical properties of asphalt mixtures under specified temperatures and loading rates, focusing on splitting failure or elastic behavior. First, Marshall specimens are placed in a constant temperature water bath at −10 °C for 1.5 h. Then, a UTM-30 material testing machine (static load ±30 kN) is used to perform the splitting test at a loading rate of 1 mm/min. Finally, the tensile strength and failure stiffness modulus of the asphalt mixture are calculated using Formulas (6) and (7) [37].

(6) $$R_{T}=0.006287P_{T}/h$$

(7) $$S_{T}=\frac{P_{T}\times (0.27+1.0\mu )}{h\times Y_{T}}$$

where *R_T_* is the splitting tensile strength, MPa. *S_T_* is the failure modulus, MPa. *P_T_* is the maximum load value during the test, N. *h* is the height of the specimen, mm. *μ* is Poisson’s ratio, typically taken as *μ* = 0.25. *Y_T_* is the total vertical deformation of the specimen under the maximum failure load, mm.

#### 2.2.5. Grey Target Decision Method

When construction materials have multiple performance indicators to consider, inconsistencies in these indicators can arise. Determining the optimal solution under multiple factors is crucial, and the grey target decision method is one approach to address this issue. The grey target decision method is based on a grey target model for decision evaluation, commonly used to assess and optimize multi-criteria schemes. The grey target represents the region of satisfactory outcomes, with a central target set on the target; the closer to the central target, the better the result. The basic idea is to identify the data closest to the target value within a set of sequences to construct a reference sequence. Each sequence forms a grey target with the reference sequence as the target center, and the distance from each data sequence to the target center, known as the target distance, is used to rank the schemes based on this distance [32,33,34]. This study assigns weights to evaluation indicators using the entropy weight method and then establishes a multi-criteria grey target decision model for calculation [36].

(1) Establishing the Sample Matrix

Assume there are *n* objects to be evaluated, and define the decision alternative set as *S* = {*S1*,*2*,…,*Sn*}. There are *m* evaluation criteria or attributes, with the set of criteria being *A* = {*A1*,*2*,…,*Am*}. The sample value of the effect of alternative *Si* on criterion *Aj* can be represented as *x*_*ij*_. Thus, the effect sample matrix of the set *S* can be represented as follows:

(8) $$X={\left(x_{ij}\right)}_{n\times m}=\left[\begin{array}{cccc} x_{11} & x_{12} & \cdots & x_{1m}\\ x_{21} & x_{22} & \cdots & x_{2m}\\ \vdots & \vdots & \vdots & \vdots \\ x_{n1} & x_{n2} & \cdots & x_{nm} \end{array}\right]$$

where *x*_*ij*_ represents the experimental value corresponding to the *j*th criterion of the *i*th alternative, where *i* = 1,2,…,*n* and *j* = 1,2,…,*m*.

(2) Establishing the Consistent Effect Measurement Matrix

To overcome the issue of different dimensions and magnitudes in the original data, dimensionless processing is required. The consistent effect measurement matrix *Y* is established according to gray target decision theory as follows:

(9) $$Y={\left(y_{ij}\right)}_{n\times m}=\left[\begin{array}{cccc} y_{11} & y_{12} & \cdots & y_{1m}\\ y_{21} & y_{22} & \cdots & y_{2m}\\ \vdots & \vdots & \vdots & \vdots \\ y_{n1} & y_{n2} & \cdots & y_{nm} \end{array}\right]$$

For cost-type (loss-type) indicators, where smaller values are better:

(10) $$y_{ij}=\frac{\max x_{j}-x_{ij}}{\max x_{j}-\min x_{j}}$$

For benefit-type (gain-type) indicators, where larger values are better:

(11) $$y_{ij}=\frac{x_{ij}-\min x_{j}}{\max x_{j}-\min x_{j}}$$

where max*x_j_* and min*x_j_* are the maximum and minimum values of the *j*th indicator among all test scenarios.

(3) Establishing the Attribute Matrix

Different evaluation criteria have varying impacts on the results. Therefore, based on the consistent effect measurement matrix, the attribute matrix is established by considering the weights of each evaluation criterion (*w*_*j*_) [34]. It is expressed as follows:

(12) $$R=w_{j}\times Y=\left[\begin{array}{cccc} r_{11} & r_{12} & \cdots & r_{1m}\\ r_{21} & r_{22} & \cdots & r_{2m}\\ \vdots & \vdots & \vdots & \vdots \\ r_{n1} & r_{n2} & \cdots & r_{nm} \end{array}\right]$$

The evaluation criterion weights (*w*_*j*_) are generally determined using methods such as the Analytic Hierarchy Process (AHP) or entropy method. This paper uses the entropy method, with the following steps:

First, define the entropy *H_j_* for the *j*th evaluation criterion:

(13) $$H_{j}=-\frac{1}{ln\;n}\sum_{i=1}^{n}f_{ij}ln(f_{ij}),1\leq j\leq m$$

(14) $$f_{ij}=\frac{y_{ij}}{\sum_{i=1}^{n}y_{ij}}$$

The weights *w_j_* are calculated according to the Equation (15).

(15) $$w_{j}=\frac{1-H_{j}}{m-\sum_{j=1}^{m}H_{j}}$$

(4) Target Center Distance

This paper adopts a hybrid elliptical decision gray target model. The target center *P* = [*p*_1_, *p_2_*, …, *p*_*n*_] ^*T*^ represents the optimal effect vector for multi-criteria gray target decision-making. The calculation formula for *p*_*j*_ is as follows:

*p_j_* = max{*r_ij_*∣1 ≤ *i* ≤ *n*, 1 ≤ *j* ≤ *m*} (16)

The calculation formula for the target center distance *L* is as follows:

(17) $$l_{i}=\sqrt{\sum_{j=1}^{m}{\left(r_{ij}-p_{j}\right)}^{2}}$$

#### 2.2.6. Statistical Analysis

Data were analyzed using SPSS 25.0. Differences between individual groups were analyzed using analysis of variance (ANOVA); mean values followed by the same letter are not significantly different as determined by ANOVA, and the differences were compared by Duncan’s range test to a significance level of 5%.

## 3. Results and Analysis

### 3.1. High-Temperature Stability

**Figure 3:**
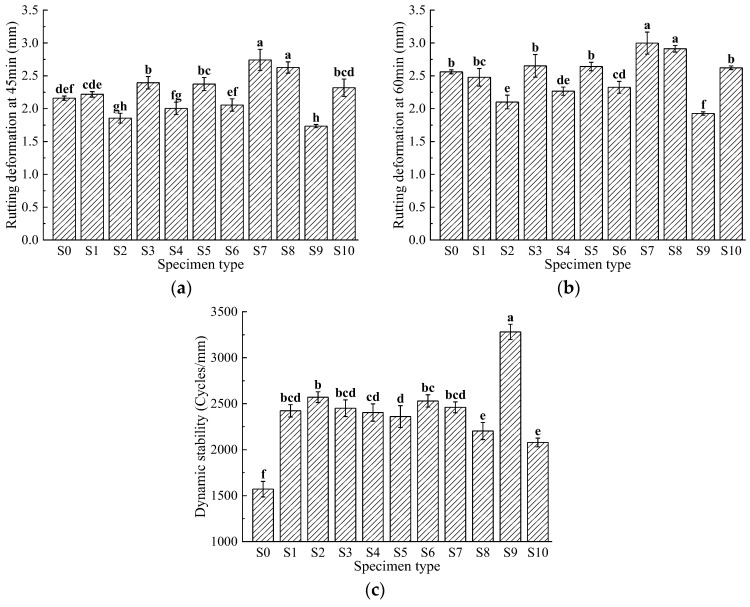
Rutting of rutting tests: (**a**) rutting deformation (45 min); (**b**) rutting deformation (60 min); (**c**) dynamic stability. Data that are significant different (*p* < 0.05) are denoted with different letters in the figure.

Due to high summer temperatures, asphalt mixtures are prone to softening, which reduces their bonding ability. After frequent vehicle traffic, the asphalt mixture pavement can develop permanent deformations such as rutting. This study evaluates the high-temperature performance of fiber-reinforced asphalt mixtures using the rutting test. The results are shown in Figure 3.

The experimental data show that basalt fiber enhances the high-temperature performance of asphalt mixtures. This is primarily due to the large specific surface area of the fibers, which allows them to adsorb a significant amount of asphalt, increasing the thickness of the asphalt film and enhancing the adhesion of asphalt to aggregates. Additionally, the basalt fiber and asphalt form a composite material that interacts with the aggregate to increase the internal friction angle, thus reducing the tendency for displacement and improving the high-temperature performance of the asphalt mixture. As illustrated in Figure 3, the rutting deformation of the basalt fiber-modified asphalt mixtures after 45 min ranges from 1.734 to 2.742 mm, and after 60 min ranges from 1.926 to 2.998 mm. The dynamic stability ranges from 2079 to 3281 cycles/mm. In comparison, the control group S0 has values of 2.16 mm, 2.561 mm, and 1571 cycles/mm. Group S9 exhibits the smallest rutting deformation and the highest dynamic stability at 3281 cycles/mm, which is 2.09 times that of the control group and represents a 1.09-fold increase.

Hao, M. et al. [39] reported that with an optimal oil-stone ratio of 5.3% and a fiber content of 0.3%, the average rutting depth after 60 min was 3.397 mm, which is greater than the maximum value of 2.998 mm found in this study. The average dynamic stability was 1428 cycles/mm, which is lower than the minimum value of 2079 cycles/mm observed in this study. In this experiment, the S9 group showed the smallest rutting deformation and the highest dynamic stability, indicating the most stable high-temperature performance. This fiber content ratio allows for efficient load transfer and dispersion to the aggregates and asphalt binder, increases the asphalt’s viscosity, and effectively reduces aggregate sliding and asphalt flow under high-temperature conditions.

Statistical analysis reveals the following: For the 45-min rutting deformation metric, groups S2, S3, S5, S7, S8, and S9 show significant differences compared to S0, indicating they significantly affect the 45-min rutting deformation. The remaining four groups show no significant differences. For the 60-min rutting deformation metric, groups S2, S4, S6, S7, S8, and S9 show significant differences compared to S0, indicating they significantly affect the 60-min rutting deformation, while the differences for the other four groups are not significant. For the dynamic stability metric, all groups show significant differences compared to S0, indicating that each group has a significant impact on dynamic stability. This is due to the bonding strength between basalt fibers and the asphalt binder, which restricts the relative movement of aggregate particles, significantly increasing the asphalt mixture’s toughness and effectively enhancing its deformation resistance. However, compared to S0, some groups (S3, S5, S7, S8) show a slight increase in rutting deformation. This may be attributed to poor dispersion of basalt fibers in these combinations, leading to negative effects. The agglomerated fibers may not provide the intended reinforcement effect and may occupy space meant for aggregates.

### 3.2. Moisture Susceptibility

**Figure 4:**
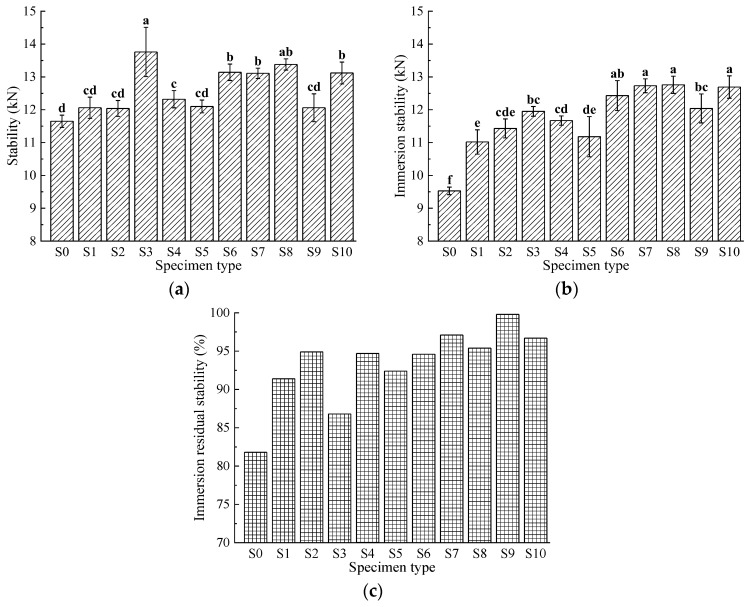
Results of Marshall tests: (**a**) stability; (**b**) immersion stability; (**c**) immersion residual stability. Data that are significant different (*p* < 0.05) are denoted with different letters in the figure.

**Figure 5:**
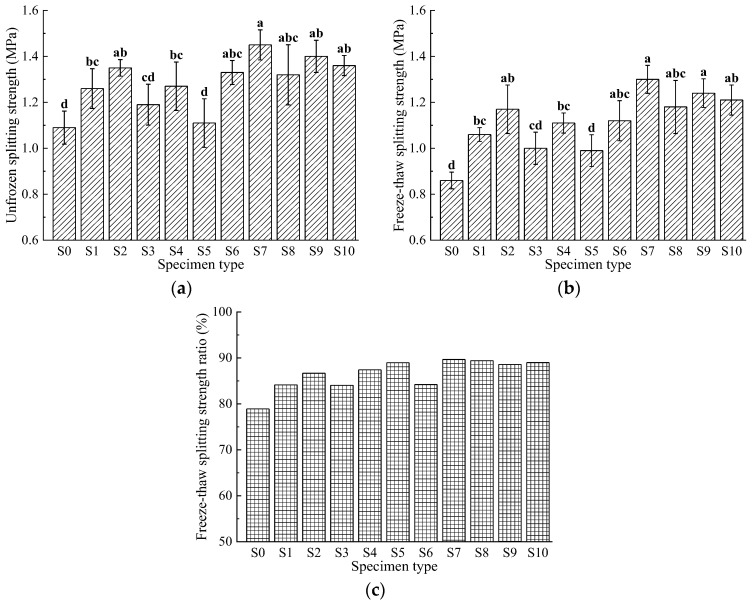
Results of freeze-thaw splitting tests: (**a**) unfrozen splitting strength; (**b**) freeze-thaw splitting strength; (**c**) freeze-thaw splitting strength ratio. Data that are significant different (*p* < 0.05) are denoted with different letters in the figure.

The infiltration of moisture into the interface between asphalt and aggregates in mixtures is a primary cause of water damage in asphalt pavements. Repeated vehicle traffic, combined with moisture entering the mixture, can lead to the detachment of asphalt from aggregate surfaces, resulting in surface defects such as raveling and potholes. Therefore, this study evaluates the water stability of fiber-reinforced asphalt mixtures using the immersion Marshall test and freeze-thaw splitting test. The results are shown in Figure 4 and Figure 5.

As shown in Figure 4, the Marshall test results indicate that as the basalt fiber gradation changes, the performance of asphalt mixtures with added basalt fiber shows some improvement. The stability of the basalt fiber-modified asphalt mixtures ranges from 12.04 to 13.76 kN, with water stability from 11.02 to 12.76 kN and water residual stability from 86.8% to 99.8%. The maximum values for these three indicators are S3 with a stability of 13.76 kN, S8 with a water stability of 12.76 kN, and S9 with a water residual stability of 99.8%. The control group S0 has values of 11.65 kN, 9.53 kN, and 81.8%, which are lower than the corresponding values of the basalt fiber-modified asphalt mixtures.

Statistical analysis reveals significant differences in water stability indicators compared to the S0 group, suggesting that these variations significantly impact water stability. However, for stability indicators, the impacts of groups S1, S2, S5, and S9 are not as pronounced, while other groups show significant effects. This is attributed to the basalt fibers’ low moisture absorption rate of less than 0.1%, which increases the optimal emulsion content in the mixture, resulting in a thicker asphalt film on the aggregates and enhanced bonding between them. Consequently, this reduces the erosion and damage caused by water to the asphalt mixtures.

Figure 5 illustrates that the freeze-thaw splitting strength and the residual strength ratio vary with the gradation of basalt fibers, and the asphalt mixtures incorporating basalt fibers demonstrated improved freeze-thaw splitting performance. This improvement is due to the dispersion of basalt fibers of various lengths within the asphalt mixture, which forms a spatial network structure capable of bearing and distributing part of the stress, thereby reinforcing the mixture and limiting crack propagation, which increases the splitting strength. The freeze-thaw splitting strength of the basalt fiber-modified asphalt mixtures ranges from 1.11 to 1.45 MPa, while the freeze-thaw splitting strength ranges from 0.99 to 1.30 MPa, and the freeze-thaw splitting strength ratio ranges from 84.03% to 89.66%. Group S7 (with basalt fiber lengths of 6 mm, 9 mm, and 12 mm at proportions of 40%, 50%, and 10%, respectively) has the maximum values, with a freeze-thaw splitting strength of 1.45 MPa, a freeze-thaw splitting strength of 1.30 MPa, and a freeze-thaw splitting strength ratio of 89.66%. Group S9 (with fiber length ratios of 6 mm, 9 mm, and 12 mm at 3:4:3) shows a freeze-thaw splitting strength of 1.40 MPa, a freeze-thaw splitting strength of 1.24 MPa, and a freeze-thaw splitting strength ratio of 88.57%. The control group S0 has a freeze-thaw splitting strength of 1.09 MPa, a freeze-thaw splitting strength of 0.86 MPa, and a freeze-thaw splitting strength ratio of 78.9%, all of which are lower than the corresponding values for the basalt fiber-modified asphalt mixtures.

Statistical analysis indicates that, compared to the S0 group, all groups except S5 show significant differences, suggesting that they all have a significant impact on both the freeze-thaw splitting strength and the freeze-thaw splitting strength ratio of the asphalt mixtures.

### 3.3. Low-Temperature Stability

**Figure 6:**
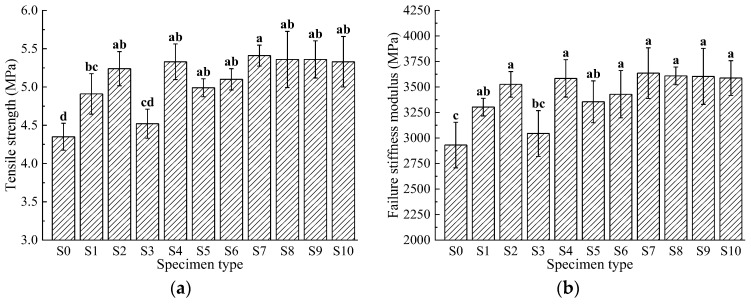
Results of low-temperature splitting tests: (**a**) tensile strength; (**b**) failure stiffness modulus. Data that are significant different (*p* < 0.05) are denoted with different letters in the figure.

Asphalt becomes stiff and brittle in cold environments, making asphalt pavements susceptible to cracking when subjected to the combined effects of thermal contraction and vehicle loading during the winter. Therefore, low-temperature splitting tests of asphalt mixtures with basalt fibers were conducted to study their crack resistance, with results shown in Figure 6.

Figure 6 demonstrates that the tensile strength and failure modulus of asphalt mixtures vary with the gradation of basalt fibers, indicating that adding basalt fibers improves the low-temperature splitting performance of asphalt mixtures. The tensile strength of the fiber-reinforced asphalt mixtures ranges from 4.52 to 5.41 MPa, and the failure modulus ranges from 3044 to 3636 MPa. Group S7 (with basalt fiber lengths of 6 mm, 9 mm, and 12 mm in proportions of 4:5:1) achieves the maximum values, with a tensile strength of 5.41 MPa and a failure modulus of 3636 MPa. Group S9 (with fiber length proportions of 6 mm, 9 mm, and 12 mm at 3:4:3) shows a tensile strength of 5.36 MPa and a failure modulus of 3604 MPa. The control group S0 has a tensile strength of 4.35 MPa and a failure modulus of 2931 MPa, both lower than those of the basalt fiber-modified asphalt mixtures.

Statistical analysis reveals that, compared to the S0 group, all groups except S3 exhibit significant differences, indicating that they all significantly affect the tensile strength and failure modulus of the asphalt mixtures. This improvement is due to the network bonding effect formed by the basalt fibers and asphalt, which inhibits the formation of cracks in the asphalt mixtures under low temperatures and load conditions, thereby enhancing both the tensile strength and failure modulus.

### 3.4. Optimization of Basalt Fiber Gradation

Based on the results from Section 3.1, Section 3.2 and Section 3.3, it is evident that different gradations of basalt fibers have varying impacts on the high-temperature performance, water stability, and low-temperature performance of asphalt mixtures. Figure 2 indicates that group S9 exhibits excellent high-temperature stability. Figure 3, Figure 4 and Figure 5 show that groups S7, S8, S9, and S10 demonstrate relatively superior performance, with statistical analysis revealing that the differences among these groups are not highly significant. Given that conventional data analysis may not effectively identify the gradation scheme with overall superior performance, a multi-objective grey target decision-making method is considered to address this issue. The calculation steps are as follows:

(1) According to the gray target decision method, the 11 experimental mix schemes form the set *S* = {*S*0, *S*1, *S*2, *S*3, *S*4, *S*5, *S*6, *S*7, *S*8, *S*9, *S*10}. The 12 experimental evaluation criteria form the set *A* = {45 min deformation, 60 min deformation, dynamic stability, stability, water stability, water residual stability, unfrozen splitting strength, frozen splitting strength, residual strength ratio, maximum load, tensile strength, failure stiffness modulus}. The decision model’s sample matrix *X* is constructed according to Equation (8) as follows:

$$X=\left[\begin{array}{llllllllllll} 2.16 & 2.56 & 1571 & 11.65 & 9.53 & 81.8 & 1.09 & 0.86 & 78.9 & 40.2 & 4.35 & 2931\\ 2.22 & 2.48 & 2423 & 12.06 & 11.02 & 91.4 & 1.26 & 1.06 & 84.13 & 49.61 & 4.91 & 3303\\ 1.86 & 2.10 & 2571 & 12.04 & 11.43 & 94.9 & 1.35 & 1.17 & 86.67 & 53.33 & 5.24 & 3526\\ 2.40 & 2.65 & 2451 & 13.76 & 11.95 & 86.8 & 1.19 & 1.00 & 84.03 & 53.08 & 4.52 & 3044\\ 2.00 & 2.27 & 2404 & 12.32 & 11.67 & 94.7 & 1.27 & 1.11 & 87.4 & 50.00 & 5.33 & 3584\\ 2.38 & 2.64 & 2360 & 12.10 & 11.18 & 92.4 & 1.11 & 0.99 & 88.94 & 49.72 & 4.99 & 3355\\ 2.06 & 2.33 & 2530 & 13.14 & 12.43 & 94.6 & 1.33 & 1.12 & 84.21 & 45.85 & 5.10 & 3429\\ 2.74 & 3.00 & 2461 & 13.11 & 12.73 & 97.1 & 1.45 & 1.30 & 89.66 & 49.27 & 5.41 & 3636\\ 2.63 & 2.91 & 2203 & 13.38 & 12.76 & 95.4 & 1.32 & 1.18 & 89.39 & 47.3 & 5.36 & 3609\\ 1.73 & 1.93 & 3281 & 12.06 & 12.04 & 99.8 & 1.40 & 1.24 & 88.57 & 48.54 & 5.36 & 3604\\ 2.32 & 2.62 & 2079 & 13.12 & 12.69 & 96.7 & 1.36 & 1.21 & 88.97 & 49.23 & 5.33 & 3588 \end{array}\right]$$

(2) According to the theory of the entropy method, among the indicators in set *A*, the 45-min deformation and 60-min deformation are cost-type indicators, which are normalized using Equation (10). The other indicators are benefit-type indicators, which are normalized using Equation (11). The consistent effect measurement matrix *Y* is obtained as follows:

$$Y=\left[\begin{array}{l} 0.57\;0.41\;0.00\;0.00\;0.00\;0.00\;0.00\;0.00\;0.00\;0.00\;0.00\;0.00\;\\ 0.52\;0.49\;0.50\;0.19\;0.46\;0.53\;0.47\;0.45\;0.49\;0.72\;0.53\;0.53\;\\ 0.88\;0.84\;0.58\;0.18\;0.59\;0.73\;0.72\;0.70\;0.72\;1.00\;0.84\;0.84\;\\ 0.34\;0.33\;0.51\;1.00\;0.75\;0.28\;0.28\;0.32\;0.48\;0.98\;0.16\;0.16\;\\ 0.73\;0.69\;0.49\;0.32\;0.66\;0.72\;0.50\;0.57\;0.79\;0.75\;0.92\;0.93\;\\ 0.36\;0.33\;0.46\;0.21\;0.51\;0.59\;0.06\;0.30\;0.93\;0.73\;0.60\;0.60\;\\ 0.68\;0.63\;0.56\;0.71\;0.90\;0.71\;0.67\;0.59\;0.49\;0.43\;0.71\;0.71\;\\ 0.00\;0.00\;0.52\;0.69\;0.99\;0.85\;1.00\;1.00\;1.00\;0.69\;1.00\;1.00\;\\ 0.11\;0.08\;0.37\;0.82\;1.00\;0.76\;0.64\;0.73\;0.97\;0.54\;0.95\;0.96\;\\ 1.00\;1.00\;1.00\;0.19\;0.78\;1.00\;0.86\;0.86\;0.90\;0.64\;0.95\;0.95\;\\ 0.42\;0.35\;0.30\;0.70\;0.98\;0.83\;0.75\;0.80\;0.94\;0.69\;0.92\;0.93 \end{array}\right]$$

(3) By substituting the consistent effect measurement matrix *Y* into Equations (12)–(15), the attribute matrix *R* can be calculated as follows:

$$R=\left[\begin{array}{l} 0.057\;0.045\;0.000\;0.000\;0.000\;0.000\;0.000\;0.000\;0.000\;0.000\;0.000\;0.000\;\\ 0.051\;0.054\;0.035\;0.026\;0.028\;0.035\;0.050\;0.035\;0.031\;0.042\;0.042\;0.042\;\\ 0.087\;0.093\;0.041\;0.024\;0.036\;0.047\;0.076\;0.054\;0.046\;0.059\;0.066\;0.067\;\\ 0.034\;0.036\;0.036\;0.132\;0.046\;0.018\;0.029\;0.024\;0.030\;0.057\;0.013\;0.013\;\\ 0.072\;0.076\;0.034\;0.042\;0.041\;0.047\;0.053\;0.043\;0.050\;0.044\;0.073\;0.073\;\\ 0.036\;0.037\;0.032\;0.028\;0.031\;0.038\;0.006\;0.022\;0.059\;0.042\;0.048\;0.048\;\\ 0.067\;0.070\;0.039\;0.093\;0.055\;0.046\;0.070\;0.045\;0.031\;0.025\;0.056\;0.056\;\\ 0.000\;0.000\;0.036\;0.091\;0.061\;0.055\;0.105\;0.076\;0.063\;0.040\;0.079\;0.079\;\\ 0.011\;0.009\;0.026\;0.108\;0.062\;0.049\;0.067\;0.055\;0.062\;0.032\;0.075\;0.076\;\\ 0.099\;0.111\;0.070\;0.026\;0.048\;0.065\;0.091\;0.066\;0.057\;0.037\;0.075\;0.076\;\\ 0.041\;0.039\;0.021\;0.092\;0.060\;0.054\;0.079\;0.061\;0.059\;0.040\;0.073\;0.074 \end{array}\right]$$

(4) Substituting the attribute matrix *R* into Formula (16), the target center *P* = [0.099, 0.111, 0.070, 0.132, 0.062, 0.065, 0.105, 0.076, 0.063, 0.059, 0.079, 0.079] ^*T*^ is obtained. Using Equation (17), the target center distances *l* for each evaluation indicator are calculated, as shown in Table 6.

Therefore, the ranking of the 11 scenarios is L_S6_ = L_S9_ < L_S10_ < L_S2_ = L_S4_ < L_S8_ < L_S7_ <L_S1_ < L_S3_ < L_S5_ <L_S0_. The optimal experimental configurations are S6, S9, and S10, with proportions of 6 mm, 9 mm, and 12 mm basalt fibers as follows: 3:4:3, 0:5:5, and 3:3:4, respectively. Following these are S2 and S4, with fiber ratios of 0:10:0 and 5:5:0. The third set includes S8 and S7, with ratios of 5:3:2 and 4:5:1. The fourth set comprises S1 and S3, with ratios of 10:0:0 and 0:0:10. S5 represents the fifth set with a ratio of 5:0:5, and the least favorable is S0 with no fiber. Combining the results of high-temperature stability tests, water stability tests, and low-temperature splitting tests reveals that the performance of specimens containing 9 mm long fibers is superior to that without them, and within equivalent scenarios, higher quantities of 9 mm fibers correspond to better performance. Thus, 9 mm fibers play a crucial role in enhancing the properties of asphalt mixtures. Asphalt mixtures exhibit better performance when the three fiber lengths are continuously proportioned, whereas performance deteriorates when the proportions of the three fiber lengths are discontinuous, and without fibers, the performance of asphalt mixtures is the poorest.

According to Table 6, the ranking of the 11 schemes is as follows: L_S6_ = L_S9_ < LS_10_ < L_S2_ = L_S4_ < L_S8_ < L_S7_ <L_S1_ < L_S3_ < L_S5_ <L_S0_. The optimal experimental schemes are S6 and S9, with basalt fiber gradations of 0:5:5 and 3:4:3 for 6 mm, 9 mm, and 12 mm fibers, respectively. The next best schemes are S10, S2, and S4, with fiber gradations of 3:3:4, 0:10:0, and 5:5:0, respectively. The third tier includes S8 and S7, with fiber gradations of 5:3:2 and 4:5:1, respectively. The fourth tier includes S1 and S3, with fiber gradations of 10:0:0 and 0:0:10, respectively. The fifth scheme is S5, with a fiber gradation of 5:0:5. The least favorable scheme is S0, which has no fiber. Since the target distances for S6 and S9 are equal, it is necessary to further evaluate these two schemes based on the experimental data from Figure 2, Figure 3 and Figure 4. For the performance indicators of the asphalt mixtures, it is evident that the performance under scheme S9 is superior.

Comparing the results from high-temperature stability tests, water stability tests, and low-temperature splitting tests, it is observed that the performance of asphalt mixtures with 9 mm fibers is better than those without 9 mm fibers. Additionally, within the same conditions, increasing the amount of 9 mm fibers improves performance. This indicates that 9 mm long fibers play a significant role in enhancing the performance of asphalt mixtures. When using a continuous gradation of fibers of three different lengths, the performance of asphalt mixtures is better. In contrast, when fibers of three different lengths are used in discontinuous gradation, the performance of the asphalt mixture is poorer, and mixtures without fibers exhibit the worst performance.

## 4. Conclusions

(1) The incorporation of basalt fibers of three different lengths into asphalt mixtures has been found to significantly improve high-temperature stability, water stability, and low-temperature performance.

(2) The performance indicators of the graded basalt fiber asphalt mixtures were superior to those of specimens without fibers. The maximum value in the dynamic stability test increased by 1.09 times, the stiffness modulus reached a maximum of 3636 MPa, the freeze-thaw splitting strength ratio was 89.66%, and the water immersion residual stability was 99.8%.

(3) Using entropy weighting to assign weights and then applying grey target decision methods, the optimal gradation scheme can be scientifically selected. Grey entropy analysis indicates that the combination of fiber content and fiber length significantly influences the performance of asphalt mixtures.

(4) Based on comprehensive testing and grey relational analysis, the optimal proportions of 6 mm, 9 mm, and 12 mm fiber lengths are recommended as 3:4:3. When designing asphalt mixtures with basalt fibers, the proportion of 9 mm fibers should be prioritized as a key control point.

## Figures and Tables

**Table 1:** Technical indicators of asphalt.

Properties	Requirements	Values
Penetration (25 °C, 5 s, 0.1 mm)	60–80	67
Ductility (15 °C, cm)	≥40	1355
Softening Point/°C	≥44	46.2
Density (g/cm^3^)		1.033

**Table 2:** Technical indicators of coarse and fine aggregates.

Aggregate	Properties	Requirements	Values
Coarse Aggregate	Apparent Relative Density	≥2.60	2.74
	Dry Relative Density		2.71
	Absolute Volume Relative Density		2.70
	Water Absorption Rate (%)	≤2.0	0.5
	Los Angeles Abrasion (%)	≤28	25.5
	Crushing Value/%	≤26	24.4
	Needle-Plate Particle Content (%)	≤15	7
Fine Aggregate	Apparent Relative Density	≥2.50	2.70
	Dry Relative Density		2.71
	Absolute Volume Relative Density		2.70
	Water Absorption (%)	≤2.0	0.5

**Table 3:** Technical indicators of mineral powder.

Properties	Requirements	Values
Apparent Density (g/cm^3^)	≥2.50	2.65
Appearance		No clumping
Water Absorption	≦1	0.2

**Table 4:** Technical indicators of basalt fiber.

Properties	Requirements	Values
Tensile Strength (MPa)	≥1200	2130
Elastic Modulus (GPa)	≥80	108
Elongation at Break (%)	2.4~3.1	2.68
Moisture Content (%)	≤0.2	0.035
Oil Absorption (%)	≥50	58

**Table 5:** Gradation of basalt fiber.

Length/mm	Fiber Content/%										
	S0	S1	S2	S3	S4	S5	S6	S7	S8	S9	S10
6	0	100	0	0	50	50	0	40	50	30	30
9	0	0	100	0	50	0	50	50	30	40	30
12	0	0	0	100	0	50	50	10	20	30	40

**Table 6:** Target center distances under different schemes.

Scheme	S0	S1	S2	S3	S4	S5	S6	S7	S8	S9	S10
Target center distances	0.27	0.17	0.13	0.18	0.13	0.20	0.11	0.16	0.15	0.11	0.12

## Data Availability

The original contributions presented in the study are included in the article, further inquiries can be directed to the corresponding author.
